# Political determinants of melanoma: Global inequities in skin cancer care

**DOI:** 10.1016/j.jdin.2026.05.006

**Published:** 2026-05-13

**Authors:** Sophia Ma, Tarek Zieneldien, Ali Aljassabi, Janice Kim, Isabella J. Tan, Jonas Willmann, Erin Jay G. Feliciano, Edward Christopher Dee, Shari Lipner, Jane M. Grant-Kels

**Affiliations:** a Johns Hopkins University School of Medicine, Baltimore, Maryland; b Department of Pharmaceutical Science, Taneja College of Pharmacy, University of South Florida. Tampa, Florida; c Michigan State University College of Osteopathic Medicine, East Lansing, Michigan; d Rutgers Robert Wood Johnson Medical School, New Brunswick, New Jersey; e Department of Radiation Oncology, University Hospital Zurich, University of Zurich, Zurich, Switzerland; f School of Medicine and Public Health, Ateneo de Manila University, Pasig City, Philippines; g Department of Medicine, NYC Health + Hospitals/Elmhurst, Icahn School of Medicine at Mount Sinai, Queens, New York; h Department of Radiation Oncology, Memorial Sloan Kettering Cancer Center, New York, New York; i Department of Dermatology, Weill Cornell Medicine, New York, New York; j Department of Dermatology, University of Connecticut School of Medicine, Farmington, Connecticut; k Department of Dermatology, University of Florida College of Medicine, Gainesville, Florida

**Keywords:** artificial intelligence, global health inequities, health insurance, melanoma, political determinants of health, regional health disparities, skin cancer, skin cancer prevention, skin of color, socioeconomic status, uninsured

## Abstract

**Background:**

Melanoma outcomes differ across countries, income settings, and skin tones, reflecting not only variable environmental and genetic risks but also upstream political determinants of health that shape exposure, access, and resources.

**Objective:**

To apply the 3-I framework (Interests, Ideas, and Institutions) to synthesize global melanoma inequities and identify actionable dermatologic implications.

**Methods:**

A narrative review of the literature (1990-2025) examined melanoma, inequities, health-system and political determinants, financing, therapies, and global and low- and middle-income country (LMIC) contexts, mapping sources to the 3-I framework.

**Results:**

Interests of governments, payers, and industry determine financing priorities, creating access gaps to immunotherapy, surgery, and pathology, particularly in LMICs and underinsured US populations. Ideas in clinical trials, artificial intelligence tools, and education underrepresent skin of color and LMIC contexts. Institutions, including segregation, colonial legacies, and centralized services, create structural barriers to timely evaluation and treatment. Examples from Australia, the US, India, and African contexts reveal recurring patterns.

**Conclusions:**

The 3-I framework (Interests, Ideas, and Institutions) identifies levers (equitable financing expansion, inclusive evidence generation, and institutional reform) through which dermatologists can promote earlier detection and equitable care via total body skin examinations for all skin tones and advocacy for covered, integrated skin cancer screening in primary care and high-risk clinic networks.

**Capsule Summary**

• Political determinants of health are central drivers of global and national inequities in melanoma prevention, diagnosis, and treatment.
• Using a 3-I framework (Interests, Ideas, and Institutions) in dermatology highlights recurring patterns and levers, such as inclusive research, equitable financing, and institutional reform, that clinicians and professional societies can target to improve melanoma outcomes worldwide.

## Introduction

Melanoma contributes substantially to global cancer burden, with rising incidence in many regions and pronounced disparities in stage at diagnosis and survival across populations. Fair-skinned individuals in high-UV environments such as Australia and New Zealand experience the highest incidence of cutaneous melanoma, whereas people with darker skin often present with more advanced disease and worse outcomes, reflecting delayed diagnosis and structural barriers rather than solely biological protection.^1^^,^^2^ In the United States, for example, Black patients with melanoma have lower 5-year survival than White patients and more frequent late-stage diagnosis; similar patterns of inequity affect other minority groups and patients in low-resource settings.^3^

**Fig 1:**
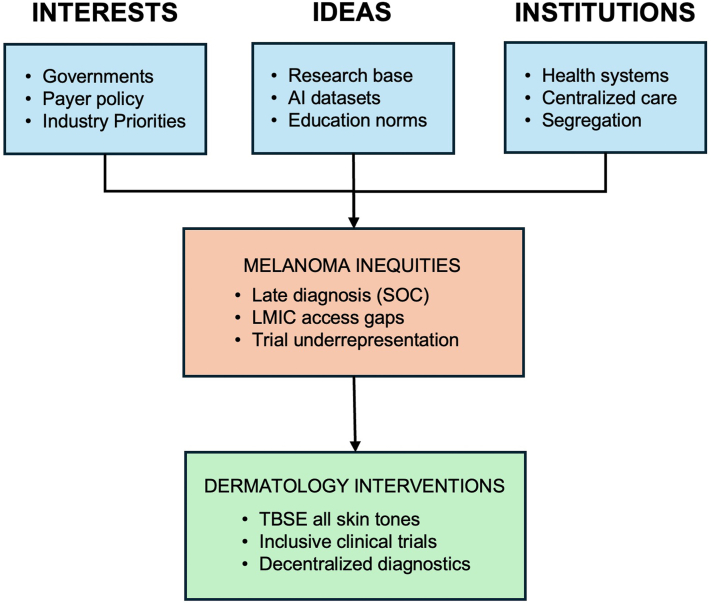
The 3-I framework (Interests, Ideas, and Institutions) organizes political determinants driving melanoma care inequities and identifies targeted dermatology interventions. Each domain contributes to late diagnosis and poor outcomes among people with skin of color and low-resource populations, whereas actionable solutions address root causes across the care continuum. *AI*, Artificial intelligence; *LMIC*, low- and middle-income country; *SOC*, skin of color; *TBSE*, total body skin exam.

The political determinants of health framework posits that health inequities stem from systems that structure relationships, distribute resources, and wield power, operating upstream of clinical encounters.^4^^,^^5^ Within political science, the 3-I model (Interests, Ideas, and Institutions) conceptualizes how stakeholders’ agendas, prevailing knowledge and values, and formal and informal rules shape policy decisions.^6^ Applying this framework to melanoma can clarify how global and national political forces drive the observed inequities in prevention, diagnosis, and treatment (Fig 1, Table I).^7^, ^8^, ^9^, ^10^, ^11^, ^12^, ^13^

This narrative review aims to (1) synthesize evidence on global melanoma inequities through the lens of the 3-I framework and (2) identify implications for dermatology practice, education, research, and policy advocacy.

**Table I:** The 3-I framework (Interests, Ideas, and Institutions) and examples from skin cancer equity

Policy decisions in skin cancer are influenced by actors' interests, ideas, and institutions		
3-I	Definition	Examples of skin cancer care
Interests	The agendas of societal groups, elected officials, civil servants, researchers, and policy entrepreneurs^12^	• In Australia, national and state governments, including Victoria, have provided sustained financial support for the SunSmart program to promote sun protection and reduce melanoma incidence among children and adolescents, illustrating how governmental priorities can sustain long-term prevention efforts.^9^^,^^10^ • In the United States, the EPA's SunWise School Program delivered sun safety education to children, with economic analysis showing each dollar invested yielded $2 to $4 in health care and productivity savings, demonstrating how cost-effectiveness aligns with governmental prevention interests.^11^
Ideas	Knowledge or beliefs about reality (eg, research findings), values or norms about priorities, or both^12^	• Melanoma clinical trials are predominantly conducted in HICs, with underrepresentation of racial and ethnic minorities and patients from LMICs, creating epistemic gaps that limit the applicability of evidence to diverse melanoma populations and reinforcing assumptions about which groups are most important to study.^8^ • High-cost immunotherapies (eg, ipilimumab, pembrolizumab, and nivolumab) have transformed metastatic melanoma survival in resource-rich settings but remain largely inaccessible in many LMICs, reflecting value priorities that favor rapid adoption of advanced therapeutics over investment in equitable access and implementation research.
Institutions	Formal and informal rules, norms, precedents, and organizational factors structuring political behavior^12^	• Historical public health communication norms, funding criteria, and media practices in many countries have prioritized fair-skinned populations and majority-language audiences in skin cancer campaigns systematically excluding underserved communities and contributing to persistent gaps in skin cancer awareness and early detection. • The INTERSUN program, led by the WHO, is a global collaboration that provides guidance on UV radiation protection for outdoor workers, children, tourists, and sunbed users, exemplifying coordinated institutional action to reduce skin cancer burden through national policy alignment and programmatic standards.^13^

*EPA*, Environmental Protection Agency; *HIC*, high-income country; *INTERSUN*, International Project on Health, Solar UV and Environmental Change; *LMIC*, low- and middle-income country; *WHO*, World Health Organization.

## Methods

A narrative review approach was used to examine the political determinants underlying global inequities in melanoma care. Literature searches were conducted in PubMed, Embase, and Scopus databases (1990-2025) using combinations of terms from 6 concept domains: (1) melanoma and skin cancer; (2) inequities and population groups; (3) political and health-system determinants; (4) financing, coverage, and access; (5) oncology therapies and trials; and (6) global and LMIC contexts.

Studies were eligible if they (1) focused on melanoma or skin cancer, and (2) examined how policies, financing mechanisms, institutional arrangements, or historical/structural factors influenced prevention, diagnosis, treatment, or outcomes; or if they offered general oncology or health-system insights with clear relevance to melanoma (eg, access to anticancer medicines or advanced systemic therapies). Because melanoma-specific evidence remains limited in some domains, findings from broader skin cancer and oncology literature were included where relevant. Commentaries without empirical or policy detail, single-patient case reports, and non-English-language articles were excluded.

After removal of duplicates, titles and abstracts were screened for relevance, followed by full-text review of potentially eligible articles. Two reviewers independently conducted the screening, with disagreements resolved by consensus. Reference lists of key publications were hand-searched to identify additional sources. Higher-quality empirical and political sources were prioritized. The final synthesis included 55 sources, which were mapped onto the 3-I framework: Interests (stakeholder agendas and incentives), Ideas (knowledge systems and values), and Institutions (rules, norms, and structural legacies), providing an evidence base for evaluating how interests, ideas, and institutions influence disparities in skin cancer prevention, diagnosis, and treatment globally and for identifying recurring national and international patterns (Table II).

**Table II:** Selected country-level system characteristics mapped to the 3-I framework (Interests, Ideas, and Institutions)

Country/setting	Key inequity/structural features	Financing and access to advanced therapy	Primary 3-I framework
Australia	Earlier-stage diagnosis is common in many urban and higher-income groups, but Indigenous populations and remote communities face later presentation and worse outcomes.^14^	Universal coverage with a public and private mix; high availability of dermatology and oncology services, and broad access to immunotherapies, though rural access gaps persist.^15^	Interests (sustained governmental prevention investment via SunSmart)
New Zealand	Marked ethnic and geographic gradients, with Māori and Pacific peoples and rural residents more likely to present with advanced disease.^16^	Publicly funded system with access to surgery and systemic therapy, but capacity constraints and travel distance contribute to inequities in timely care.^17^	Institutions (centralized services creating geographic barriers)
United States	Black and other minoritized groups have a lower incidence but a higher likelihood of late-stage diagnosis and poorer survival; disparities are linked to differences in access, quality of care, and structural factors.^18^	Fragmented multi-payer landscape; access to advanced therapies depends heavily on insurance type, co-sharing, and prior authorization, with underinsured patients facing major barriers.^19^	Interests (payer/manufacturer priorities shape therapy access)
India	Late presentation is frequent; rural residents and low-income groups have limited access to dermatology, pathology, and oncology services.^20^	Public spending on health remains relatively constrained; PM-JAY and state schemes expand coverage for inpatient cancer care but do not fully address out-of-pocket costs or geographic barriers.^21^	Interests (national financial risk protection reforms)
Selected African settings (eg, South Africa, Ghana)	Patients often present with advanced disease; historical segregation and centralization of tertiary services create long travel distances and high indirect costs for rural and marginalized groups.^22^	Limited pathology and oncology capacity in many public systems; high-cost immunotherapies and targeted agents are rarely available in public sectors, with very constrained access in private markets.^23^	Institutions (colonial/apartheid legacies, service centralization)
United Kingdom	Socioeconomic and regional gradients in stage at diagnosis and survival, with residents of more deprived areas experiencing worse outcomes.^24^	The National Health Service provides universal coverage, but workforce shortages, referral bottlenecks, and regional variation in cancer service capacity affect timely access to dermatology and oncology.^25^	Institutions (NHS workforce/ referral system design)

*NHS*, National Health Service; *PM-JAY*, Pradhan Mantri Jan Arogya Yojana.

### Interests: Stakeholders, financing, and regulatory priorities

Interests encompass the agendas and incentives of governments, payers, industry, professional societies, and advocacy groups, which together shape the allocation of resources and policy attention to melanoma.^6^^,^^7^

#### Financing and coverage for melanoma care

In many LMICs, constrained public health spending and limited prepayment mechanisms result in high out-of-pocket costs for cancer care, which can delay melanoma diagnosis and restrict access to surgery, pathology, and systemic therapy.^26^, ^27^, ^28^, ^29^ A global analysis of anticancer medicines documented substantial gaps in the availability and affordability of oncology drugs, especially in low- and middle-income settings, highlighting how financing decisions and market structures restrict access to effective oncology, including therapies used in advanced melanoma, such as immunotherapy.^26^^,^^27^

India illustrates how national interests in financial risk protection can partially mitigate these barriers. Historically low public health expenditure contributed to catastrophic costs for cancer care, but the Ayushman Bharat Pradhan Mantri Jan Arogya Yojana, a large publicly funded health insurance program, has expanded coverage for inpatient services, including oncologic care, for low-income families. Evaluations from Punjab show that benefit package reforms under Pradhan Mantri Jan Arogya Yojana increased utilization of covered services and reduced financial hardship, demonstrating how government priorities and benefit design can reshape access to cancer surgery and systemic therapy in a way that is relevant for melanoma care.^30^

In high-income countries, financing structures also mediate who receives advanced melanoma treatments. In the United States, racial and ethnic minority groups and individuals with lower incomes are more likely to be uninsured or underinsured; they face greater barriers to specialist consultation and oncology services.^8^^,^^30^, ^31^, ^32^, ^33^, ^34^ At the same time, high-cost therapies such as checkpoint, BRAF, and MEK inhibitors have transformed survival for patients with metastatic melanoma but remain strongly dependent on insurance coverage, co-payment levels, and prior authorization requirements.^35^, ^36^, ^37^ These dynamics align the interests of payers and manufacturers with certain patient populations while de-prioritizing others.^29^

#### Prevention programs and governmental agendas

The distribution of political attention to melanoma prevention also reflects stakeholder interests. In Australia, national and state governments, including the Victorian Government, have provided sustained financial and policy support for the SunSmart program, which has promoted sun protection behaviors and is associated with improvements in population-level sun protection and reductions in melanoma incidence in younger cohorts.^9^^,^^10^ In the United States, the Environmental Protection Agency’s SunWise School Program provided sun safety education for children, and an economic evaluation estimated that each dollar invested generated 2 to 4 dollars in savings from reduced health care costs and productivity loss, illustrating how economic arguments can align with governmental interests in primary prevention.^11^

Conversely, where competing health priorities and limited budgets dominate the agenda, skin cancer prevention may receive lower priority, and investments in shade infrastructure, occupational protections, and targeted community outreach are less likely to be funded, as seen in New Zealand.^7^^,^^28^^,^^38^^,^^39^ These patterns demonstrate that inequities emerge in part from how governments and other stakeholders weigh prevention against short-term demands and from whose risk is seen as politically salient.^7^^,^^38^

### Ideas: Knowledge systems, representation, and perceived risk

Ideas include the knowledge, beliefs, and values that shape how melanoma risk is conceptualized, which populations are prioritized in research and policy, and what interventions are considered legitimate.^28^^,^^40^

#### Evidence production and epistemic inequities

The majority of randomized controlled oncology clinical trials are disproportionately conducted in high-income countries and often underrepresent patients from LMICs and racial and ethnic minority groups, particularly in skin cancer trials.^8^^,^^41^ Analyses of melanoma trials indicate that minority enrollment remains low compared with disease burden, limiting the applicability of trial findings to diverse at-risk populations^8^ Lower-income patients are also less likely to participate in cancer clinical trials, reflecting financial and logistical barriers that further skew the evidence base toward advantaged groups.^42^ These patterns contribute to what has been termed “epistemic inequities,” in which the experiences and outcomes of marginalized populations are underrepresented in the knowledge that guides policy and practice.^28^^,^^31^^,^^32^

A similar pattern is seen in dermatologic artificial intelligence. In a curated dermatologic image dataset, an artificial intelligence diagnostic tool demonstrated differential performance across skin tones, with reduced accuracy in images of darker skin, including lesions relevant to melanoma detection.^43^ Educational materials and atlases have also historically emphasized melanoma in lighter skin, with fewer images of acral, mucosal, or non-sun-exposed melanomas in people with skin of color (SOC).^31^^,^^44^, ^45^, ^46^, ^47^ When clinicians are trained mainly on fair-skinned images, melanomas in darker skin, often presenting on the palms, soles, nail beds, or mucosal surfaces, are more likely to be missed or diagnosed late.^45^, ^46^, ^47^

#### Values and prioritization of high-cost therapies

Prevailing ideas about what constitutes “cutting-edge” cancer care have prioritized rapid development and adoption of high-cost targeted therapies and immune checkpoint inhibitors, particularly in high-resource settings.^28^^,^^35^ These agents, including ipilimumab and subsequent generations of checkpoint inhibitors, have transformed the prognosis of metastatic melanoma in clinical trials and routine practice.^5^^,^^36^ However, assessments of anticancer drug access show that these therapies are often unavailable, unaffordable, or delayed in many LMICs, where competing budgetary pressures constrain adoption.^26^, ^27^, ^28^

The global research agenda has similarly emphasized discovery and evaluation of novel therapeutics over implementation research, context-appropriate prevention, and service delivery innovations in resource-constrained settings.^28^^,^^48^ These value choices reinforce a cycle in which evidence and investment are concentrated in contexts and populations that already benefit from robust melanoma services, whereas the structural causes of late presentation and limited access elsewhere receive less attention.^28^^,^^41^^,^^48^

### Institutions: Historical legacies, health system design, and structural barriers

Institutions comprise the formal and informal rules, norms, precedents, and organizational structures that govern political behaviors and shape health systems.^6^^,^^32^^,^^40^^,^^49^

#### Segregation, colonial legacies, and spatial distribution of services

Historical patterns of racial segregation and structural racism have left enduring marks on the geography of cancer care. In the United States, research on multiple cancers has shown that neighborhoods with histories of redlining, a discriminatory practice that restricted investment in predominantly Black and other minority communities, have higher odds of late-stage diagnosis, reflecting cumulative disadvantage in access to health services and broader social determinants of health.^27^^,^^49^^,^^50^

In South Africa and other countries with colonial and apartheid legacies, tertiary cancer centers and specialized dermatologic services remain concentrated in historically advantaged urban areas, despite legal reforms.^49^ Patients from rural and historically marginalized communities often face long travel distances and financial barriers to reach centers with biopsy, pathology, and specialized cancer care facilities, including those necessary for skin cancer treatment.^49^ Similar centralization of cancer services in major cities has been documented in other LMICs, including India, Ghana, and the Philippines, creating structural delays in diagnosis and treatment for peripheral populations.^28^^,^^48^^,^^51^

#### Integration of skin cancer care into existing platforms

The organization of health services also influences whether melanoma is detected early or remains neglected. A survey of HIV treatment centers across 29 countries found substantial gaps in the availability of skin cancer diagnostic and treatment services, particularly in low- and middle-income settings, despite the elevated skin cancer risk among immunosuppressed patients.^51^ Because HIV clinics often serve as consistent points of contact with the health system for large numbers of adults in low-resource settings, these broad skin cancer service gaps likely limit early melanoma detection opportunities as well. Not integrating basic skin cancer screening and referral pathways into these platforms reflects institutional design choices that constrain access to timely diagnosis.^48^^,^^51^

#### Institutional responses to UV exposure and occupational risk

At the global level, the International Project on Health, Solar UV and Environmental Change program, led by the World Health Organization, provides guidance to governments on strategies to reduce UV-related skin cancer burden, including melanoma, including recommendations for protecting outdoor workers, children, sunbed users, and tourists.^13^ National and local programs that integrate shade provision into built-environment planning and public health agendas, such as shade initiatives in New South Wales, Australia, demonstrate how institutional action can structurally reduce UV exposure over time.^39^ However, occupational studies continue to show that many outdoor workers, such as male Hispanic day laborers in the United States, experience frequent sunburns and have limited access to sun protection, reflecting gaps in the implementation of such institutional protections.^52^

Institutional norms within health care organizations also shape melanoma outcomes. Limited emphasis on full-body skin examination in nondermatology settings, inadequate inclusion of SOC images in educational resources, and underrepresentation of minoritized groups in dermatology training collectively contribute to delayed melanoma recognition among patients with darker skin.^45^, ^46^, ^47^^,^^53^ These institutional factors intersect with interests and ideas, reinforcing a multilayered system of barriers across the melanoma care continuum.

### Implications for dermatology practice, education, and policy

Applying the 3-I framework to melanoma highlights several actionable implications for dermatologists, health systems, and professional organizations.

In clinical practice, dermatologists can promote equitable prevention by supporting policies that extend sun protection and UV-safety programs to high-risk, underserved groups and by encouraging sun protection from childhood through adulthood. At the same time, clinicians should acknowledge that many melanomas in SOC are less UV-related and therefore require focused efforts on recognition and examination of acral, nail, and mucosal sites. In SOC patients, acral lentiginous and mucosal melanomas, which are not primarily linked to UV exposure, constitute a substantially greater proportion of all melanomas than in White patients. Not only do they characteristically arise on sun-protected sites, but they also frequently arise in areas of chronic inflammation or scarring.^45^^,^^46^^,^^54^ Many individuals from underserved populations remain unaware that they are at risk of skin cancer, including melanoma, and lack knowledge of warning signs; clinicians should offer full-body skin examinations to patients of all skin tones and provide culturally and linguistically appropriate education on melanoma risk and early signs, although it should be noted that visual skin examinations remain an evidence-limited, context-dependent recommendation.^53^

In education, dermatology residency and fellowship programs can integrate robust SOC content throughout the curriculum rather than confining it to isolated sessions, ensuring that teaching images, simulation exercises, and case discussions include diverse presentations of melanoma, particularly acral and mucosal subtypes. Embedding structured teaching on political and structural determinants of melanoma disparities can help trainees connect epidemiologic patterns to upstream causes, including financing arrangements, regulatory decisions, and historical segregation. CME can explicitly assess competencies in recognizing melanoma across skin tones and anatomic sites, counseling patients from varied cultural backgrounds, and understanding how institutional and policy environments shape access to timely diagnosis and treatment. Professional organizations can curate open-access image libraries and clinical tools that focus on melanoma in SOC and in resource-constrained settings, countering epistemic inequities in the visual and clinical resources that currently guide practice.

In research and policy, clinicians can advocate for clinical trials and observational studies that set enrollment targets for underrepresented populations, systematically report race, ethnicity, and skin phototype, and measure structural factors such as insurance status, geography, and income alongside tumor characteristics and treatment. Partnerships with investigators and institutions in LMICs as equal collaborators, rather than peripheral recruitment sites, are important for generating context-relevant evidence on prevention, diagnosis, and treatment pathways and for aligning research agendas with locally defined priorities. Professional societies can work with policymakers, payers, and patient advocacy groups to expand financial protection for melanoma surgery, pathology services, and systemic therapies, including immunotherapies and targeted agents, to reduce expenditures for low-income patients and improve access to effective treatment. Policy advocacy can also prioritize integration of basic melanoma screening and referral into existing platforms such as HIV programs, primary care, and occupational health clinics, alongside investments in shade infrastructure, occupational protections for outdoor workers, teledermatology, and decentralization of diagnostic capacity to regional centers, thereby addressing institutional design choices that currently limit early detection and comprehensive melanoma care in underserved settings.

## Conclusion

Melanoma inequities systematically stem from political determinants structured by interests, ideas, and institutions, informed by both melanoma-specific evidence and broader skin cancer and oncology literature. Dermatologists can target **I**nterests through advocacy for coverage expansion, **I**deas through inclusive clinical trials and SOC curricula, and **I**nstitutions through decentralized diagnostics and integrated screening platforms. Professional societies can curate diverse image libraries, establish trial diversity targets, and forge equitable partnerships with LMIC institutions, particularly in resource-constrained settings. This 3-I framework (Interests, Ideas, and Institutions) equips global dermatology to address structural melanoma disparities and measure progress across diverse health systems.

## Conflicts of interest

None disclosed.
